# Analysis of tobacco consumption, before and during the COVID-19 pandemic in Peru

**DOI:** 10.18332/tid/149905

**Published:** 2022-05-27

**Authors:** Akram Hernández-Vásquez, Fabriccio J. Visconti-Lopez, Rodrigo Vargas-Fernández

**Affiliations:** 1 Centro de Excelencia en Investigaciones Económicas y Sociales en Salud, Vicerrectorado de Investigación, Universidad San Ignacio del Loyola, Lima, Peru; 2 Facultad de Ciencias de la Salud, Universidad Peruana de Ciencias Aplicadas, Lima, Peru; 3 Facultad de Ciencias de la Salud, Universidad Científica del Sur, Lima, Peru

**Keywords:** tobacco consumption, COVID-19, Peru

**Dear Editor,**

Tobacco consumption is a global health problem that has caused more than 200 million deaths in the last 30 years^1^. A decrease in global prevalence has been reported in the last two decades due to cost-effective strategies to reduce morbidity and mortality by this cause^2^. Despite this, 80% of tobacco consumption is observed in low- and middle-income countries^3^. Several countries report a decrease in tobacco consumption during the COVID-19 pandemic^4,5^. However, to date, it remains unknown whether these findings are similar in South American countries.

Therefore, we sought to determine the change in the prevalence of current tobacco use, before and during the COVID-19 pandemic in the Peruvian adult population following the methodology described by Barrera-Nuñez et al.^5^. The prevalence of tobacco consumption in the last 30 days was estimated by sex, based on the 2019 and 2020 Demographic and Family Health Surveys. Prevalence ratios were obtained for the change in tobacco consumption during the study period, stratified according to age and socioeconomic variables.

A total of 61206 (2019: 31035; 2020: 30171) respondents were included in the analysis. The prevalence of current tobacco consumption was 11.2% in 2019 (men: 18.7%; women: 4.1%) and 8.0% in 2020 (men: 13.5%; women: 2.8%). Compared to 2019, the largest reductions in tobacco consumption in 2020 were reported in men, among individuals aged ≥60 years (-60%), in those with a higher education (-42%), persons belonging to the rich wealth quintile (-28 %), and residing in rural areas (-41%), small cities (-33%) and in the Highlands (-45%), while women only in some subgroups showed decreases in tobacco consumption (Table 1).

**Table 1:** Prevalence ratios of current tobacco consumption in Peruvian adults between 2019 and 2020

*Characteristics*	*Overall*	*Men*	*Women*
	*PR (95% CI)*	*PR (95% CI)*	*PR (95% CI)*
**Age** (years)			
18–29	0.76 (0.66–0.87)*	0.75 (0.65–0.88)*	0.81 (0.59–1.12)
30–59	0.70 (0.63–0.78)*	0.73 (0.65–0.83)*	0.58 (0.43–0.79)*
≥60	0.58 (0.43–0.78)*	0.55 (0.40–0.77)*	0.69 (0.36–1.33)
**Education level**			
Up to Primary	0.67 (0.55–0.81)*	0.68 (0.56–0.84)*	0.59 (0.34–1.00)
Secondary	0.73 (0.65–0.83)*	0.76 (0.67–0.86)*	0.72 (0.51–1.02)
Higher	0.67 (0.58–0.76)*	0.68 (0.58–0.79)*	0.62 (0.46–0.84)*
**Wealth quintile^a^**			
Poorer	0.72 (0.62–0.84)*	0.71 (0.61–0.82)*	1.18 (0.68–2.05)
Poor	0.68 (0.58–0.81)*	0.71 (0.60–0.85)*	0.52 (0.30–0.91)*
Medium	0.77 (0.64–0.92)*	0.78 (0.65–0.95)*	0.71 (0.44–1.12)
Rich	0.64 (0.53–0.78)*	0.67 (0.55–0.83)*	0.58 (0.40–0.85)*
Richer	0.71 (0.58–0.88)*	0.72 (0.57–0.91)*	0.69 (0.46–1.04)
**Area of residence**			
Urban	0.71 (0.64–0.78)*	0.73 (0.66–0.81)*	0.66 (0.52–0.83)*
Rural	0.69 (0.60–0.79)*	0.68 (0.59–0.78)*	0.91 (0.55–1.51)
**Place of residence^b^**			
Countryside	0.69 (0.60–0.79)*	0.68 (0.59–0.78)*	0.91 (0.55–1.51)
Town	0.70 (0.60–0.81)*	0.74 (0.63–0.87)*	0.50 (0.34–0.72)*
Small city	0.66 (0.58–0.77)*	0.67 (0.57–0.78)*	0.66 (0.48–0.90)*
Capital	0.74 (0.63–0.86)*	0.76 (0.64–0.91)*	0.69 (0.50–0.96)*
**Natural area**			
Jungle	0.77 (0.68–0.87)*	0.79 (0.69–0.90)*	0.73 (0.49–1.10)
Highlands	0.65 (0.55–0.75)*	0.64 (0.55–0.75)*	0.65 (0.44–0.98)*
Coast	0.71 (0.63–0.80)*	0.73 (0.64–0.83)*	0.67 (0.51–0.87)*

Data source: Demographic and Family Health Survey (ENDES) 2019 and 2020.

* p<0.05.

a The wealth quintile is a variable provided by the ENDES obtained from the characteristics of the dwelling and assets in the household.

b The place of residence is classified as capital (capital cities and cities with more than 1 million inhabitants), small city (more than 50000 inhabitants) and town (other urban areas), and countryside corresponds to rural areas. PR: prevalence ratio; indicates the comparison of the year 2020 with respect to 2019 for each category of the variable of interest. CI: confidence interval. The dependent variable was tobacco consumption in the last 30 days. All estimates took into account the ENDES sample design and were adjusted for age. ENDES: is a national population-based survey that provides information on the health status of the Peruvian population. Further details and access to public databases can be found at: https://proyectos.inei.gob.pe/endes/documentos.asp

Similar to what was reported in other countries^4,5^, in Peru there was a decrease in the prevalence of current tobacco consumption in men and for some subgroups in women during 2020. These findings could be attributed to the emotional responses (fear, anxiety and sadness) generated by the dissemination of epidemiological studies, news and health recommendations that linked smoking with a higher risk of severity and death from COVID-19^6,7^, a reduction in income, limitation of social environments, and mobility restrictions^6^, resulting in a lower consumption compared to 2019.

While the reduction in smoking during the pandemic is encouraging, it is also the right time for the measures established in the World Health Organization Framework Convention to return to the public agenda and to further efforts to achieve global control of the tobacco epidemic.

## Data Availability

Data sharing is not applicable to this article as no new data were created.
